# Prevalence of macular disorders assessed by OCT in adults 45 years and older from Parintins - the Brazilian Amazon Region Eye Survey (BARES)

**DOI:** 10.1186/s40942-026-00834-1

**Published:** 2026-05-09

**Authors:** Sung E. S. Watanabe, Adriana Berezovsky, Arthur G. Fernandes, Bruna F. Marianelli, João Marcello Furtado, Marcela Cypel, Paulo Henrique Morales, Marcos J. Cohen, Cristina C. Cunha, Márcia H. Mitsuhiro, Galton C. Vasconcelos, Mauro Campos, Nívea N. Ferraz, Paula Y. Sacai, Jacob M. Cohen, Sergio Muñoz, Rubens Belfort Jr., Solange R. Salomão

**Affiliations:** 1 Departamento de Oftalmologia e Ciências Visuais, Escola Paulista de Medicina, Universidade Federal de São Paulo – UNIFESP, Rua Botucatu, 806. Vila Clementino, São Paulo, SP CEP 04023-062 Brazil; 2 Departamento de Oftalmologia, Otorrinolaringologia e Cirurgia de Cabeça e Pescoço, Faculdade de Medicina de Ribeirão Preto, Universidade de São Paulo – USP, Ribeirão Preto, SP Brazil; 3 Departamento de Oftalmologia e Otorrinolaringologia, Faculdade de Medicina, Universidade Federal de Minas Gerais (UFMG), Belo Horizonte, MG Brazil; 4 Divisão de Oftalmologia, Departamento de Cirurgia, Faculdade de Medicina, Universidade Federal do Amazonas – UFAM, Manaus, Brazil; 5 Departamento de Salud Publica, Universidad de La Frontera, Temuco, Chile

**Keywords:** Spectral-domain optical coherence tomography, Population-based eye survey, Macular disorders prevalence, Retinal abnormalities, Vision impairment, Subclinical retinal disease detection

## Abstract

**Background:**

To provide population-level estimates of OCT-detected macular pathology in adults living in the Brazilian Amazon region.

**Methods:**

Population-based, cross-sectional study. Eligible participants were residents aged ≥ 45 years identified through cluster sampling (20 clusters: 14 urban and 6 rural) in Parintins City, Amazonas, Brazil. Ophthalmic examinations included visual acuity testing, biomicroscopy, fundoscopy, and subjective refraction. A subset of eligible participants underwent spectral-domain OCT (SD-OCT; iVue-100, Optovue) using macular mapping protocols. OCT images were graded for predefined abnormalities involving the vitreoretinal interface, inner retina, outer retina/retinal pigment epithelium (RPE)/choroid, and ganglion cell complex (GCC). Generalized estimating equations (GEE) were used to evaluate factors associated with any OCT abnormality. Statistical significance was set at *p* < 0.05.

**Results:**

Of 2,384 eligible individuals, 2,041 (85.7%) were examined and 588 (28.8%) underwent OCT (1,176 eyes), of which 1,069 eyes (90.9%) were gradable. OCT macular abnormalities were detected in at least one eye in 180 participants (30.6%). Overall, outer retinal layer changes were most frequent, followed by GCC thinning and epiretinal membrane. OCT abnormalities were independently associated with older age and lower educational level. Structural abnormalities were identified in 340 (31.8%) eyes; of these, 208 (19.5%) showed changes also detectable on dilated fundus examination, whereas 132 (12.3%) had a clinically normal funduscopic appearance. Clinically notable lesions were detected in 46 (4.3%) eyes, including signs of late AMD in 27 (2.5%) eyes, diabetic maculopathy in 6 (0.6%), lamellar macular hole in 6 (0.6%), full-thickness macular hole in 3 (0.3%), and central serous chorioretinopathy in 4 (0.4%) eyes. Among 774 eyes clinically normal at fundoscopy, OCT revealed subclinical disease in 127 (16.4%).

**Conclusions:**

In this underserved Amazonian population, approximately one-third of gradable eyes showed OCT-detected macular abnormalities, many of them subclinical on fundoscopy. Diabetic maculopathy, choroidal neovascular membranes, and late AMD showed very low prevalence and limited epidemiologic weight in this setting. Incorporating OCT into population-based surveys enhances detection and refines burden estimates of subclinical retinal disease and vision-threatening conditions in aging populations.

## Introduction

Retinal diseases are one of the leading causes of blindness and vision impairment in adults 50 years and older worldwide. More recent estimates for blindness and moderate to severe vision impairment showed 8.06 million cases of age-related macular degeneration (AMD) and 3.81 million cases of diabetic retinopathy (DR) [1, 2]. In Brazil, retinal disorders were the main cause of severe visual impairment and blindness (47%), followed by cataract (40%) in a population-based study conducted in 2004–2005 in adults 50 years and older performed in low-income urban areas of São Paulo city [3]. In a second survey, conducted in 2014–2015 in the city of Parintins (Brazilian Amazon region), including adults 45 years and older, retinal diseases were detected by a comprehensive ophthalmic in-person examination, including dilated fundus exam, in 15.28% of participants, regardless of the visual acuity status [4]. 

Optical coherence tomography (OCT) is a non-invasive imaging modality mainly used in ophthalmology to provide high-resolution imaging of the retina [5]. OCT brought significant contributions in the field of diagnosis and monitoring of a variety of retinal diseases, such as AMD, central serous chorioretinopathy, macular hole, vitreomacular interface syndrome, and diabetic maculopathy among others [6]. 

The Brazilian Amazon Region Eye Survey (BARES) was a population-based study aimed at investigating the prevalence and causes of distance and near visual impairment, as well as blindness, among older adults living in Parintins, a city in the Amazon Region. This area has distinct geographic, demographic, and sociocultural characteristics that set it apart from other regions of Brazil, particularly the Southeast, which has been the focus of more research in the past. The lack of health information regarding this specific population can be attributed, in part, to difficulties in access and the operational challenges faced during the study [7]. 

In population-based studies aiming to determine the cause of visual impairment and blindness in areas with limited access to eye care, the OCT is a valuable tool to enhance the precision and proper referral for those with retinal diseases. There is a scarcity of population-based studies for those living in underprivileged and remote geographic areas such as the Brazilian Amazon.

Main findings from the BARES study had been previously published, addressing vision status [8], prevalence and causes of visual impairment and blindness for distance [9] and near [10], prevalence of pterygium [11], cataract surgery complications [12], impact of pterygium on central corneal thickness (Dotto) [13], prevalence of ocular findings in the anterior and posterior segment regardless visual acuity [4] and normative values for macular thickness by OCT [14]. 

The purpose of this study was to determine the prevalence of OCT abnormalities in adults 45 years and older from Parintins city, Brazilian Amazon Region.

## Methods

### Study design and population

BARES was a population-based, cross-sectional study designed to provide practical estimates of the prevalence of near and distance vision impairment and blindness among non-institutionalized residents of Parintins city. Fieldwork was conducted during September 2013 to May 2015. OCT was included in the study to clarify the ocular causes of vision impairment and blindness. However, due to logistic and geographic barriers in the Amazon region, OCT was performed only on a subset of participants. The study was approved by the Committee on Ethics for Research of *Universidade Federal de São Paulo* and *Universidade Federal do Amazonas* and followed the Declaration of Helsinki guidelines. Written informed consent was obtained from all participants.

Cluster sampling, based on geographically defined census sectors, identified residents aged ≥ 45 years living in 20 randomly selected clusters (14 urban and 6 rural) in Parintins, located on the margins of the Amazon River, Amazonas State, Brazil. Eligibility was determined via door-to-door surveys, and participants were invited to attend a clinical ophthalmic examination. Inclusion criteria included being at least 45 years old on the day of the household visit and residing in one of the selected clusters for at least the past 6 months. Further details on the study area, the identification of eligibility, visual acuity testing, and eye examinations are available elsewhere [7]. 

As previously described [7], the ethnic composition of Amazonian Brazilians results from a historical process starting with indigenous groups, followed by immigration of White Europeans (mainly Portuguese), African descendants, and Brazilians from other regions, particularly the Northeast during the rubber boom [15–17]. The IBGE defines color or race as the self-reported skin color by the interviewed person including five categories: White, Black, multiracial (mixed White, African, or Indigenous ethnicity), Yellow (Asians) and Indigenous [18]. The 2010 IBGE census reported that 64.8% of inhabitants in the Brazilian Amazon Region identified as multiracial (“pardo”), followed by 25.1% of as White skin color, 7.5% of as Black skin, 1.6% as Indigenous, and 1.1% as Asians [19, 20]. 

### Ocular examination

The ophthalmic examination included uncorrected visual acuity and acuity with presenting correction, measured using a retro-illuminated logarithm of the minimum angle of resolution (logMAR) tumbling E chart at 4 m. Best-corrected visual acuity (BCVA) was determined after autorefraction, followed by subjective refraction, conducted by an ophthalmologist. Biomicroscopy was performed before and after pupil dilation, focusing on lens status, classified as normal, phakic with cataracts, pseudophakic, pseudophakic with posterior capsule opacity, aphakic, aphakic with posterior capsule opacity, or undetermined. Intraocular pressure (IOP) was measured using applanation tonometry under topical anesthesia after administering 0.5% proxymetacaine hydrochloride eye drops. A fundus examination was also conducted under pupil dilation using 1% tropicamide eye drops.

### OCT images acquisition protocol

SD-OCT scans were performed using the iVue − 100 SD-OCT device (Optovue Inc., Fremont, California, USA) after pupil dilation. The system’s image acquisition rate was 25,000 A-scans/s, with frame rates between 256 and 1024 A-scans/frame. Optical resolution was 5 μm in depth. The scan pattern used was the “Retina Map”, which is a 6 × 6 mm raster pattern of 13 horizontal line scans (6 mm long, 512 A-scans each), plus additional 7 horizontal line scans (1024 A-scans) within central 1.5 mm vertical zone. Image quality was assessed using the signal strength index (SSI), which measures reflected light intensity (scoring from 0 to 100). Scans with poor quality were excluded from analysis (for instance, eyes with relevant media opacities and/or optical distortion from severe pterygium). For instance, images with segmentation failures, algorithm errors, motion artifacts, poor focus, lack of centering, or SSI below 50 were omitted.

The macular thickness measurement method was described elsewhere [14]. The Early Treatment Diabetic Retinopathy Study (ETDRS) subfields were used to assess full retinal thickness across nine regions, including parafoveal, perifoveal, nasal, superior, inferior, and temporal areas. Thickness measurements included both inner retinal boundaries (from the internal limiting membrane to the outer limit of the inner plexiform layer) and outer retinal boundaries (from the outer limit of the inner plexiform layer to the retinal pigment epithelium). In total, 29,174 A-scans were analyzed, with a higher sampling density at the central fovea. The EMM5 protocol consisting of a primary macular mapping scan, was used to generate average foveal thickness (1 mm in diameter, centered on the fovea) as well as full, inner, and outer retinal thickness maps. The ganglion cell complex (GCC), defined as the combined thickness from the inner limiting membrane to the inner plexiform layer, was measured using 7 × 7 mm raster scans aligned to the macular region.

### OCT qualitative analysis

All OCT scans were qualitatively evaluated by experienced retinal specialists (SESW, BFM) to identify structural abnormalities based on established tomographic criteria. Images were systematically reviewed in a masked fashion, and abnormalities were classified according to their anatomical location (vitreoretinal interface, inner retina, outer retina, retinal pigment epithelium, and choroid). Reflectivity patterns, contour alterations, and layer integrity were used as primary descriptors for each lesion.

For analytical purposes, OCT-detected abnormalities were categorized into five major groups: (1) Epiretinal membrane (ERM) [21]; (2) Vitreomacular interface disorders (VMI), including vitreomacular traction, lamellar macular hole, and full-thickness macular hole [22, 23]; (3) Ganglion cell complex thinning (GCCT) [24]; (4) Outer retinal changes (ORC), encompassing drusen, early and late age-related macular degeneration (AMD), retinal pigment epithelium (RPE) atrophy, and photoreceptor layer atrophy; [25–28] (5) Other abnormalities, including intraretinal cysts, inner retinal atrophy or disorganization, choroidal folds, and central serous chorioretinopathy [29–31]. 

All findings were documented in a standardized data collection sheet. Discrepancies between graders were resolved by consensus review. This approach ensured consistent qualitative classification of tomographic abnormalities, enabling subsequent statistical analyses.

### Statistical analysis

Statistical analyses were conducted using STATA/SE version 14.0 (StataCorp, College Station, TX). Descriptive statistics were summarized using frequency distributions for categorical variables and means with standard deviations for continuous variables. Confidence intervals (CIs) for prevalence estimates and odds ratios (ORs) were calculated with adjustment for the cluster sampling design. Statistical significance was defined as *p* ≤ 0.05. Participant-level analyses were performed using multivariable logistic regression models, with the individual as the unit of analysis. These models were used to examine associations between demographic factors and the presence of any OCT-detected abnormality (binary outcome defined at the participant level). Eye-level analyses were conducted to evaluate specific OCT-detected abnormalities (epiretinal membrane, vitreomacular interface disorders, outer retinal changes, ganglion cell complex thinning, and other abnormalities). Because both eyes from the same individual were included, generalized estimating equations (GEE) with an exchangeable correlation structure and robust standard errors were used to account for intra-individual correlation between fellow eyes. Given that analyses of individual OCT abnormalities were prespecified and addressed clinically distinct outcomes, formal adjustment for multiple comparisons was not applied. These analyses were interpreted in the context of predefined hypotheses and clinical plausibility.

## Results

A total of 2,384 eligible persons were identified, 2,041 (85.7%) were examined, and 588 (28.8%) individuals had OCT assessment (1,176 eyes). Out of these, 1,069 (90.9%) eyes had gradable OCT scans. The age ranged from 45 to 90 years (mean = 59.51 ± 10.00 years), 275 males (46.77%) and 313 (53.23%) females. Table 1 presents the demographic characteristics of the overall study cohort and the OCT subgroup, stratified by sex, age, educational level, and residence.

**Table 1:** Demographic characteristics of the total study population and the subgroups with and without OCT assessment

	Total	OCT assessment	No OCT assessment
Sex			
Female	1041 (51.00)	313 (53.23)	728 (50.10)
Male	1000 (49.00)	275 (46.77)	725 (49.90)
Age			
45-54	790 (38.71)	209 (35.54)	581 (39.99)
55-64	646 (31.65)	210 (35.71)	436 (30.01)
65-74	351 (17.20)	107 (18.20)	244 (16.79)
75+	254 (12.44)	62 (10.54)	192 (13.21)
Education			
None	237 (11.61)	41 (6.97)	196 (13.49)
<Primary	546 (26.75)	139 (23.64)	407 (28.01)
Primary	577 (28.27)	149 (25.34)	428 (29.46)
Secondary+	681 (33.37)	259 (44.05)	422 (17.82)
Residence			
Urban	1180 (57.81)	522 (88.78)	658 (45.29)
Rural	861 (42.12)	66 (11.22)	795 (54.71)
Total	2041 (100.00)	588 (100.0)	1453 (100.00)

There were no significant differences in sex between individuals who underwent the OCT examination and those who did not (*p* = 0.200). However, individuals who underwent OCT were younger (*p* = 0.025), had higher levels of education (*p* < 0.001), and were more frequently residents of urban areas (*p* < 0.001).

Of the 588 participants with OCT data, 180 individuals (30.6%) demonstrated at least one structural abnormality in a single eye, whilst 80 participants (13.6%) exhibited bilateral abnormalities. Multivariate logistic regression analysis demonstrated statistically significant associations between the presence of OCT abnormalities and advancing age and rural residence. Individuals aged 55 to 64 old were 1.52 times more likely to have an OCT abnormality than those aged 45 to 54 years old (OR = 1.52; 95%CI: 1.09–2.11; *p* = 0.014); individuals aged 65 to 74 old were 2.06 times more likely to have an OCT abnormality than those aged 45 to 54 years old (OR = 2.06; 95%CI: 1.39–3.06; *p* < 0.001), and individuals aged 75 years and more were 2.62 times more likely to have an OCT abnormality than those aged 45 to 54 years old (OR = 2.62; 95%CI: 1.63–4.21; *p* < 0.001). Individuals living in rural areas were 2.07 times more likely to have an OCT abnormality than those living in urban areas (OR = 2.07; 95%CI: 1.40–3.06; *p* < 0.001). Moreover, a significant difference in the prevalence of OCT abnormalities was observed between those with higher levels of education and those with no formal schooling. Individuals with secondary level education or better were 0.55 times more likely to have an OCT abnormality than those with no formal education (OR = 0.55; 95%CI: 0.32–0.94; *p* = 0.030) (Table 2).

**Table 2:** Prevalence of OCT-detected abnormalities and associated risk factors

	OCT assessed	OCT abnormalities	Odds Ratio (95%CI)	p-value
	N (%)	N (%)		
Sex				
Female	313 (53.23)	99 (31.63)	Ref	---
Male	275 (46.77)	81 (29.45)	0.83 (0.64 – 1.08)	0.160
Age				
45-54	209 (35.54)	41 (19.62)	Ref	---
55-64	210 (35.71)	66 (31.43)	1.52 (1.09 – 2.11)	0.014
65-74	107 (18.20)	43 (40.19)	2.06 (1.39 – 3.06)	<0.001
75+	62 (10.54)	30 (48.39)	2.62 (1.63 – 4.21)	<0.001
Education				
None	41 (6.97)	20 (48.78)	Ref	---
<Primary	139 (23.64)	58 (41.73)	1.01 (0.60 – 1.72)	0.962
Primary	149 (25.34)	47 (31.54)	0.76 (0.44 – 1.30)	0.313
Secondary+	259 (44.05)	55 (21.24)	0.55 (0.32 – 0.94)	0.030
Residence				
Urban	522 (88.78)	147 (28.16)	Ref	---
Rural	66 (11.22)	33 (50.00)	2.07 (1.40 – 3.06)	<0.001
Total	588 (100.0)	180 (30.61)		

Following individual OCT assessment (1069 eyes), structural abnormalities were identified in 260 (24.32%) eyes. Of these, 85 eyes (32.69%) had moderate to severe visual impairment or blindness (best-corrected visual acuity [BCVA] ≤ 0.5 logMAR), whereas 175 eyes (67.31%) did not. In contrast, among eyes without OCT-detected abnormalities, 87 eyes (10.75%) had moderate to severe visual impairment or blindness, while 722 eyes (89.25%) had BCVA better than 0.5 logMAR. The presence of OCT-detected abnormalities was significantly associated with worse visual acuity (χ² test, *p* < 0.001).

Among the 1,069 eyes analyzed, outer retinal changes were the most prevalent, identified in 114 eyes (10.66%) followed by ganglion cell complex thinning in 84 eyes (7.86%), epiretinal membrane in 44 eyes (4.12%), vitreomacular interface disorders in 13 eyes (1.22%), and other abnormalities in 28 eyes (2.62%). Table 3 presents the distribution of OCT abnormalities according to visual acuity status.

**Table 3:** Distribution of OCT findings according to visual acuity status

	No VI		Mild VI		Moderate VI		Severe VI		Blindness		**Total**	
	20/20–20/32		20/40 − 20/63		20/80 − 20/200		< 20/200 − 20/400		< 20/400			
	RE	LE	RE	LE	RE	LE	RE	LE	RE	LE		
ERM	13	13	3	9	3	1	0	1	0	0	43	22%
VMI	6	4	0	0	0	0	1	1	0	0	12	6%
ORC	21	34	8	10	7	3	0	3	2	1	89	46%
GCC	13	11	5	2	2	0	0	0	0	0	33	17%
Others*	6	2	2	3	0	0	1	1	0	1	16	8%
Total	59	64	18	24	12	4	2	6	2	2	193	100%

Legend: VI= visual impairment; ERM=epiretinal membrane; VMI= vitreomacular interface disorders; ORC= outer retinal changes; GCC= ganglion cell complex thinning. Other: intraretinal cysts, inner retinal atrophy or disorganization, choroidal folds, and serous retinal epithelium detachment

In the cohort of 1,069 gradable eyes, conditions prominent in population-based studies – such as late age-related macular degeneration, diabetic retinopathy, and myopic macular degeneration – did not exhibit high prevalence. Specifically, OCT identified clinically notable abnormalities in 46 eyes (4.3%) including signs of late AMD in 27 (2.5%) eyes, 6 (0,6%) eyes with diabetic maculopathy, lamellar macular hole in 6 (0.6%) eyes, full-thickness macular hole in 3 (0.3%) eyes and central serous chorioretinopathy in 4 (0.4%) eyes.

Generalized Estimating Equations (GEE) analysis revealed a significant and increasing association between the presence of ERM and age, with prevalence rising markedly in older age groups. Compared to participants aged 45–54 years, those aged 55–64 had significantly increased odds of ERM (OR = 6.19, 95% CI: 1.79–21.48; *p* = 0.004), which further increased among individuals aged 65–74 (OR = 8.09, 95% CI: 2.16–30.37; *p* = 0.002), and this risk was further elevated in participants aged ≥ 75 years (OR = 13.29, 95% CI: 3.28–53.85, *p* < 0.001). These results indicate a strong age-related gradient, with the likelihood of OCT-detected ERM increasing markedly in older individuals.

Outer retinal changes including both early and advanced AMD, demonstrated statistically significant higher prevalence in males (OR = 1.60, 95% CI: 1.05–2.42, *p* = 0.028), those aged ≥ 75 years (OR = 2.17, 95% CI: 1.09–4.35, *p* = 0.027) and rural residence (OR = 2.51, 95% CI: 1.47–4.26, *p* < 0.001). On the other hand, higher educational level was associated with lower odds of outer retinal changes (OR = 0.30, 95% CI: 0.14 − 0.65, *p* = 0.002). Similarly, ganglion cell complex thinning was significantly less prevalent in males (OR = 0.47, 95% CI: 0.28–0.77, *p* = 0.003), whereas the odds of thinning were significantly higher in participants aged 65 years or older—particularly those aged 65–74 years (OR = 2.68, 95% CI: 1.33–5.39, *p* = 0.006) and ≥ 75 years (OR = 3.82, 95% CI: 1.67–8.78, *p* = 0.027), as well as among individuals residing in rural areas (OR = 2.51, 95% CI: 1.47–4.26, *p* = 0.030).

Signs of prior cataract surgery were identified in 72 eyes (6.73%). The prevalence of OCT abnormalities was substantially higher among post-surgical eyes, with 36 eyes (50.0%) exhibiting at least one abnormality. In 997 eyes without evidence of previous cataract extraction, at least one OCT change was found in 224 eyes (22.5%). The most common findings in operated eyes were epiretinal membrane (31.82%) followed by vitreomacular interface abnormalities (23.08%), and outer retinal changes (14.04%).

### Fundus findings vs. OCT

Among the 1,069 eyes examined, 774 (72.40%) had a normal fundus appearance on clinical examination, while macular abnormalities were observed in 295 eyes (27.60%). Among eyes with clinically apparent macular alterations, 45.08% also showed structural abnormalities on OCT. Conversely, among the 774 eyes with normal fundus examination, 127 (16.41%) exhibited subclinical changes detectable only by OCT. It is important to note that some eyes presented more than one OCT abnormality.

## Discussion

This study represents the first population-based assessment of macular abnormalities using OCT in adults aged 45 years and older in the Brazilian Amazon region. Our findings demonstrate that 24.32% of examined eyes harbored structural changes. This highlights the incremental diagnostic value of OCT in detecting early or subclinical macular abnormalities that are frequently missed by conventional ophthalmoscopy [32, 33]. The integration of OCT into epidemiological surveys therefore provides a more accurate estimate of the true burden of retinal disease in underserved populations [34]. 

The prevalence of moderate to severe visual impairment or blindness was substantially higher among eyes with OCT-detected abnormalities compared to those without structural changes (32.69% vs. 10.75%, *p* < 0.001). Among the spectrum of abnormalities detected, outer retinal changes—primarily early and late AMD —were the most common (10.66%), followed by ganglion cell complex (GCC) thinning (7.86%) and epiretinal membrane (4.12%). When restricting the analysis to early AMD, the prevalence was 37 (3.5%) eyes among 1,069 gradable eyes. The high prevalence of outer retinal changes reflects the expected age-related susceptibility to degenerative processes such as AMD, whereas the detection of GCC thinning underscores the capacity of OCT to identify early neurodegenerative events [35, 36]. GCC analysis is particularly relevant, as thinning of these inner retinal layers may indicate underlying glaucomatous damage or other optic neuropathies that remain undetected in routine population screenings [36, 37]. Epiretinal membranes and vitreomacular interface disorders, though less prevalent, were also noteworthy findings, suggesting that tractional changes contribute meaningfully to the overall macular morbidity in this community. In our population-based sample, we found early AMD in 3.5% and any AMD in 6.3%, which is lower than rates commonly reported in large global summaries [38–41]. Potential explanations for these findings include our cohort of adults from 45 years upward, with fewer participants > 70 years, the ethno-demographic profile of the Amazonian population, with substantial ethnic admixture that may modulate genetic risk, and we classified disease mainly by OCT, which may under-detect early, pigmentary changes better seen on color photographs; moreover, only a subset had OCT. As in other settings, late AMD was uncommon.

Sociodemographic analyses revealed significant inequities. Increasing age was strongly associated with abnormal findings, in line with the known pathophysiology of degenerative retinal and vitreomacular diseases [35, 42, 43]. However, lower educational attainment also emerged as a significant risk factor, suggesting that social determinants of health may contribute to delayed diagnosis, reduced awareness, and poorer access to eye care services [44–46]. The higher prevalence of abnormalities in rural residents further reinforces the impact of geographic disparities in access to specialized ophthalmic evaluation [47, 48]. Interestingly, sex was not consistently associated with the overall prevalence of OCT abnormalities, although stratified analyses indicated that men exhibited higher rates of outer retinal changes and GCC thinning [49–51]. These differences warrant further exploration to assess the role of behavioral, environmental, and biological factors in modulating susceptibility.

From a clinical perspective, the OCT abnormalities documented here warrant emphasis because they help explain visual loss that might otherwise be misattributed to cataract, a common occurrence in older adults. Recognizing macular diseases early refines the differential diagnosis, informs pre-operative counseling, and aligns expectations for cataract surgery outcomes. Strikingly, we observed a higher prevalence of OCT abnormalities among eyes with previous cataract surgery, particularly epiretinal membranes and vitreomacular interface conditions. This association may reflect postoperative alterations in vitreoretinal adhesion, as well as the possibility that individuals undergoing surgery represent a subgroup already at greater risk of ocular morbidity [52, 53]. Given the high prevalence of cataract surgery in older populations, these findings underscore the importance of vigilant postoperative retinal monitoring, especially in low-resource settings where follow-up care may be inconsistent. The use of OCT in cataract-operated eyes with suspected visual abnormalities plays a crucial role in identifying underlying macular abnormalities, such as cystoid macular edema or subclinical maculopathy, which may not be evident on biomicroscopy alone and can significantly impact postoperative visual outcomes [12]. 

From a public health standpoint, these findings provide compelling evidence for the incorporation of OCT into population-based eye health surveys. Traditional epidemiological approaches relying solely on visual acuity and ophthalmoscopy underestimate the prevalence of retinal disease by overlooking subclinical or early-stage lesions [54]. In our study, a notable proportion of eyes with a normal fundus appearance (16.41%) also exhibited structural changes on OCT. These findings confirm the added diagnostic value of OCT in identifying subtle retinal changes that are not apparent on conventional ophthalmoscopy [32–34]. Incorporating OCT into population-based surveys can therefore enhance the detection of vision-threatening retinal conditions that may otherwise go unnoticed during standard clinical evaluations. Beyond its diagnostic precision, OCT also increases sensitivity in detecting subtle retinal pathology and offers the advantage of identifying early and potentially treatable lesions. Importantly, image acquisition and interpretation can be automated and transmitted by telemedicine technology, reducing the dependency on physician-based grading and enabling large-scale screening of silent retinal diseases using advanced technology. This makes OCT an attractive tool for population-level triage strategies in low-resource settings, where early detection could translate into significant gains in preventable blindness.

Some limitations should be acknowledged. First, OCT was performed in only a subset of participants due to logistic and geographic barriers, resulting in a subgroup with higher representation of urban residents and individuals with higher educational attainment. This differential participation may have introduced selection bias and limits the generalizability of prevalence estimates to the overall Parintins population. Accordingly, the reported prevalence of OCT-detected abnormalities should be interpreted as descriptive of the OCT-assessed subgroup rather than population-level estimates. However, associations between demographic factors and OCT abnormalities were evaluated using multivariable models within the examined subgroup, supporting the internal validity of observed relationships. In low- and middle-income settings such as Brazil, incorporating OCT devices into population-based studies imposes considerable logistical and financial challenges. However, ongoing technological innovations and the rise of AI-based screening tools are gradually lowering costs and expanding access to high-resolution retinal imaging in large-scale epidemiologic research [55]. 

Second, the cross-sectional design precludes assessment of longitudinal progression, hindering conclusions on the natural history of detected abnormalities. Moreover, while expert grading ensured reliability, the absence of multimodal imaging restricted the ability to fully characterize certain lesions. Future studies should incorporate longitudinal follow-up, larger and more representative samples, and integration of multimodal imaging to refine disease classification and prognostic assessment. Expanding access to portable OCT devices and artificial intelligence–based image analysis could further enhance population screening strategies, particularly in underserved regions with limited specialist availability.

In conclusion, in this underserved Amazonian population, approximately one third of gradable eyes showed OCT-detected macular abnormalities, many of them subclinical on fundoscopy. Diabetic maculopathy, choroidal neovascular membranes, and late AMD showed very low prevalence and limited epidemiologic weight in this setting. Incorporating OCT into population-based surveys improves detection and refines burden estimates of subclinical retinal disease and vision-threatening conditions in aging populations.

## Data Availability

The datasets used and/or analyzed during the current study and available from the corresponding author on reasonable request.
